# Identification and verification of heat shock protein 60 as a potential serum marker for colorectal cancer

**DOI:** 10.1111/j.1742-4658.2011.08385.x

**Published:** 2011-12

**Authors:** Céline Hamelin, Emilie Cornut, Florence Poirier, Sylvie Pons, Corinne Beaulieu, Jean-Philippe Charrier, Hader Haïdous, Eddy Cotte, Claude Lambert, Françoise Piard, Yasemin Ataman-Önal, Geneviève Choquet-Kastylevsky

**Affiliations:** 1 Immunoproteomics Laboratory, Department of BiomarkersbioMérieux, Marcy l’étoile, France; 2 Department of Anatomo-pathology, Dijon University HospitalFrance; 3 University of Paris 13CNRS CSPBAT/LBPS, Bobigny, France; 4 Department of Clinical TrialsbioMérieux, Marcy l’étoile, France; 5 Department of Surgical Oncology, Lyon Sud University HospitalPierre Bénite, France; 6 University of Lyon IEMR 3738, Oullins, France; 7 Immunology Laboratory, Saint-Etienne University Hospital, Center for Health Engineering UMR-CNRS 5148 LPMG and IFR 143 INSERM IFRESISFrance

**Keywords:** 2D-DIGE, colorectal cancer, HSP60, marker validation, serum biomarker

## Abstract

Colorectal cancer (CRC) is a major public health issue worldwide, and novel tumor markers may contribute to its efficient management by helping in early detection, prognosis or surveillance of disease. The aim of our study was to identify new serum biomarkers for CRC, and we followed a phased biomarker discovery and validation process to obtain an accurate preliminary assessment of potential clinical utility. We compared colonic tumors and matched normal tissue from 15 CRC patients, using two-dimensional difference gel electrophoresis (2D-DIGE), and identified 17 proteins that had significant differential expression. These results were further confirmed by western blotting for heat shock protein (HSP) 60, glutathione-*S*-transferase Pi, α-enolase, T-complex protein 1 subunit β, and leukocyte elastase inhibitor, and by immunohistochemistry for HSP60. Using mAbs raised against HSP60, we developed a reliable (precision of 5–15%) and sensitive (0.3 ng·mL^−1^) immunoassay for the detection of HSP60 in serum. Elevated levels of HSP60 were found in serum from CRC patients in two independent cohorts; the receiver-operating characteristic curve obtained in 112 patients with CRC and 90 healthy controls had an area under the curve (AUC) of 0.70, which was identical to the AUC of carcinoembryonic antigen. Combination of serum markers improved clinical performance: the AUC of a three-marker logistic regression model combining HSP60, carcinoembryonic antigen and carbohydrate antigen 19-9 reached 0.77. Serum HSP60 appeared to be more specific for late-stage CRC; therefore, future studies should evaluate its utility for determining prognosis or monitoring therapy rather than early detection.

## Introduction

With an incidence of more than 1.2 million new cases and 600 000 deaths per year, colorectal cancer (CRC) is a major public health issue worldwide [1]. Currently, mass screening relies principally on fecal occult blood tests [2,3], and the reference standard for diagnosis confirmation is colonoscopy, an invasive method that causes major morbidity in 0.3% of subjects [4,5]. Diagnosis and treatment of CRC at an early stage of cancer development considerably improves the chances of survival; patients diagnosed at an advanced stage have a rather poor prognosis. In fact, disease stage at the time of diagnosis is still the main prognostic factor for CRC.

Surgical resection is the recommended treatment for most CRC patients; stage III patients will receive adjuvant chemotherapy following surgery, which improves survival probability at 5 years [6]. The utility of adjuvant chemotherapy in stage II patients is still subject to debate, and its use in this population is not recommended, although there is clear evidence that it would be helpful for a subgroup of patients with stage II disease [7]. One of the important needs in CRC management is the identification of stage II patients who may benefit from adjuvant chemotherapy. Up to 40–50% of CRC patients will develop advanced disease over time, despite treatment efforts [8]. Another clinical need is the surveillance of patients following completion of therapy, in order to detect recurrence of disease as early as possible. Monitoring therapy in advanced disease is also beneficial [9].

Carcinoembryonic antigen (CEA) was one of the first serological tumor markers to be discovered, and has contributed significantly to the acceptance of tumor markers as aids in making clinical decisions. Today, after 30 years of clinical research, it is well established that CEA should not be used for screening or early detection of CRC, and that it has some utility for determining prognosis as well as monitoring advanced disease, in association with clinical history. The unique clinical indication in which CEA is consensually recommended by different expert groups is postoperative surveillance; there is solid cumulative evidence demonstrating its utility for this specific purpose [9,10].

Several other serum markers, such as carbohydrate antigen 19-9 (CA19-9), carbohydrate antigen 242 and tissue inhibitor of metalloproteinases type 1, have been developed, and are being evaluated for various clinical uses in CRC management (reviewed in [9]). Regrettably, none has the required diagnostic performance to be considered for screening or early detection purposes. These serum markers may contribute to determining prognosis or monitoring therapy, but high-powered, controlled studies are still needed to assess their added value. Therefore, there is a need for new CRC biomarkers that will satisfactorily meet one or several of the clinical needs discussed above. Serum markers are preferred over tissue or stool-based assays, especially for screening and monitoring purposes, which require repeat testing. Blood-based tests have better acceptance and provide increased patient compliance.

The aim of our study was to identify such candidate protein markers and investigate their possible clinical use. We used a proteomics strategy relying on 2D-gel-based discovery in tissue and further confirmation of potential candidates in serum. Recent examples in the literature show that similar approaches can successfully yield novel serum biomarkers for CRC, such as nicotinamide-*N*-methyltransferase [11], proteasome activator complex subunit [12], S100A8, and S100A9 [13,14]. To date, none of these tumor markers has been completely clinically evaluated and has shown utility. Given the diversity of clinical needs in CRC management, the small number of candidate serum biomarkers, and the low success rate of clinical utility assessments, it is necessary to identify novel, additional tumor markers and to determine the clinical indications in which they may have an added value. We compared colonic tumors and matched normal mucosa from CRC patients, using 2D difference gel electrophoresis (2D-DIGE), and identified 17 proteins that had significant differential abundance. Among them, heat shock protein (HSP) 60 was reported to be actively secreted by tumor cells [15], and its expression in tissue was correlated with tumor grade and progression [16,17]. Thus, it appeared to be the best candidate for evaluation as a potential serum marker for CRC. We followed the multistep biomarker discovery and validation process proposed by Rifai *et al.*, which involves candidate discovery, qualification, verification, assay optimization and biomarker validation phases [18]. Our results are reported using the terminology proposed in this process. Using a well-characterized and robust research immunoassay specifically designed for the detection of HSP60 in serum, we successfully completed the verification phase, and were able to show, for the first time, that HSP60 levels are more frequently increased in the serum of CRC patients than in healthy controls. Serum HSP60 seemed to be more specific for late-stage cancer, so it might be better suited for disease monitoring than for early detection.

## Results

### Identification of differentially expressed proteins with 2D-DIGE

**Fig. 1:**
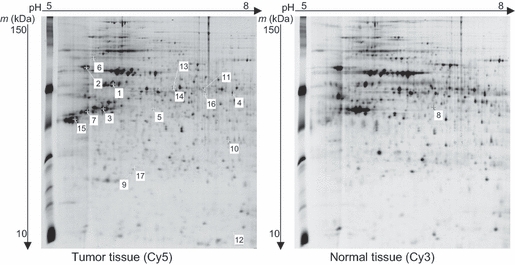
Representative 2D-DIGE maps of colonic tissue (patient 6). Soluble proteins extracted from colon tumor (Cy5) and matched normal tissue (Cy3) were labeled with the indicated dyes, mixed with Cy2-labeled internal standard, and subjected to IEF on pH 5-8 IPG strips. Protein samples were then separated on large-format 7.7–16.5% gradient SDS/PAGE gels. Molecular mass separation is 150–10 kDa (top to bottom). Numbered spots indicate proteins that have statistically significant differential expression between tumor tissue and adjacent normal mucosa (fold-change over 1.5 and *P* < 0.05 with Wilcoxon signed-rank test). MALDI-TOF MS identification results for these spots are shown in Table 2.

**Fig. 2:**
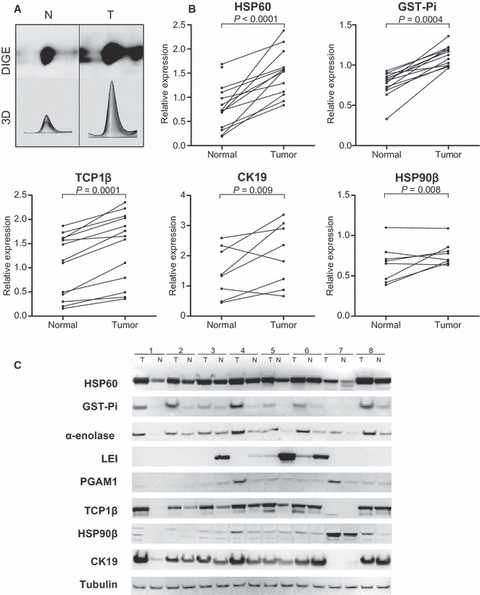
Western blot qualification of differentially expressed protein spots. (A) 2D-DIGE image and corresponding 3D simulation of the HSP60 spot in a matched tissue sample. N, normal tissue; T, tumoral tissue. (B) Relative expression of HSP60, GST-Pi, TCP1β, CK19 and HSP90β in paired CRC samples analyzed by 2D-DIGE. Relative expression corresponds to the spot volume determined with imagemaster 2d-platinium software, transformed into logarithm base 2, and normalized with the corresponding spot volume of the internal standard image (Cy2). Comparisons were performed with the Wilcoxon signed-rank test. (C) Western blot analysis of protein expression in eight independent tissue sample pairs. Tubulin was used as loading control.

Colonic tumor and matched normal mucosa were obtained from 15 patients undergoing surgical resection (Table 1). Epithelial cells were purified from each tissue specimen, and total protein extracts were prepared. For each patient, expression was compared between protein extracts of tumor and normal epithelial cells with 2D-DIGE analysis, including a dye swap replicate between Cy3 and Cy5 to avoid labeling bias [19,20]. The internal standard was a cytoplasmic protein extract from Caco-2 cells labeled with Cy2 dye. A total of 30 well-resolved gels (two for each patient) were obtained, and on each gel ∼ 800 protein spots were detected in a pI range of 5–8 (Fig. 1). After background subtraction, in-gel normalization, and removal of artefact spots, the matching rate of each internal standard gel and the master gel (Cy2) reached over 90%. Following matching, image analysis was carried out to compare the median ratio of protein abundance between paired colon tumor tissues and adjacent normal mucosa in the 2D-DIGE maps (Fig. 2A). Relative protein expression, which corresponds to log_2_-transformed, normalized spot volumes, is shown in Fig. 2B for some selected spots. Protein spots that were above the 1.5-fold-change threshold were tested for statistical significance. Among 17 spots that were determined to be significantly different between tumor and normal colon mucosa, 16 were upregulated in adenocarcinoma and one was downregulated (Fig. 1; Table 2).

**Table 1:** Clinical data of colon cancer patients used in 2D-DIGE and western blot analyses. UICC, Union for International Cancer Control

Patient no.	Age (years)	Sex	Global staging (UICC)	Tumor localization	TNM staging
2D-DIGE					
1	84	F	III	Right colon	T4N2M0
2	71	F	III	Sigmoid	T3N1M0
3	61	M	III	Right colon	T3N2M0
4	75	M	II	Left colon	T3N0M0
5	73	M	II	Left colon	T3N0M0
6	73	M	II	Right colon	T3N0M0
7	57	M	II	Right colon	T3N0M0
8	71	M	II	Right colon	T3N0M0
9	73	M	III	Left colon	T3N1M0
10	79	M	II	Left colon	T3N0M0
11	61	M	IV	Left colon	T2N0M1
12	88	F	II	Right colon	T3N0M0
13	65	F	III	Left colon	T1N1M0
14	79	M	III	Sigmoid	T3N2M0
15	78	M	III	Right colon	T3N1M0
Western blot					
1	82	M	II	Left colon	T4N0M0
2	76	M	IV	Right colon	T4N2M1
3	72	M	IV	Right colon	T4N1M1
4	62	M	IV	Left colon	T3N1M1
5	79	F	II	Right colon	T3N0M0
6	59	M	III	Transverse colon	T3N1M0
7	77	M	IV	Right colon	T3N0M1
8	73	M	IV	Left colon	T2N0M1

**Table 2:** Identification of proteins with differential expression in colon cancer by MALDI-TOF MS. Accession number in SWISS-PROT protein database. Spot number reported in Fig. 1. *n*, number of patient samples in which the spot was identified. Fold-change ratio: a positive ratio indicates increased abundance in colon carcinoma, and a negative ratio indicates a decrease. *P-*value of Wilcoxon test applied to *n* paired 2D-DIGE analysis results. Protein score: amino acid sequence coverage. Previous report: previously reported as differential expression in CRC (normal versus tumor); M indicates that the difference was observed between metastatic and nonmetastatic colon cancer; + or − signs are used when there is controversy, and indicate the differential expression that was reported in the associated study

Recommended name	Accession number	Spot number	Molecular mass (kDa)	pI	*n*	Fold-change ratio	*P*-value	Protein score	Sequence coverage (%)	Previous report
60-kDa heat shock protein, mitochondrial	P10809	1	61.1	5.6	15	3.25	< 0.0001	267	57.1	21,23,27
78-kDa glucose-regulated protein	P11021	2	72.3	4.9	15	1.60	0.028	301	47.4	[27] −
Actin, cytoplasmic 1	P60709	3	41.7	5.5	8	3.96	< 0.0001	229	60.8	21,27
α-Enolase	P06733	4	47.2	7.7	8	1.59	0.002	259	62.1	11,13,21–23
Aminoacylase-1	Q03154	5	45.9	5.7	15	1.78	0.003	228	40.7	
Heat shock protein 90β	P08238	6	83.3	4.8	8	1.59	0.008	183	47.6	[42] M
Keratin, type I cytoskeletal 19	P08727	7	44.1	4.9	8	1.54	0.009	186	45.5	21
Leukocyte elastase inhibitor	P30740	8	42.7	5.9	11	−2.26	0.006	250	50.9	[28] +
Peroxiredoxin-2	P32119	9	21.9	5.6	15	1.67	0.041	164	68.7	
Phosphoglycerate mutase 1	P18669	10	28.8	6.8	15	2.82	0.002	159	70.8	[23] −, [27] +, [42] +
Pre-mRNA-processing factor 19	Q9UMS4	11	55.2	6.0	15	1.71	0.0001	129	44.6	
Protein S100-A8	P05109	12	10.8	6.6	15	2.01	< 0.0001	148	81.7	13
T-complex protein 1 subunit α	P17987	13	60.3	5.7	15	2.45	0.040	71	13.7	
T-complex protein 1 subunit β	P78371	14	57.5	5.3	15	1.71	0.0001	145	34.8	
Tropomyosin β-chain	P07951	15	32.9	4.5	8	1.79	0.0002	77	34.2	[25,26] M
Elongation factor 1γ	P26641	16	50.0	6.3	11	1.85	0.008	213	43.8	28
Glutathione-*S*-transferase Pi	P09211	17	23.2	5.3	15	1.53	0.0004	239	62.2	[26] M, [28]

MS identification of protein spots was carried out by using replicate gels with 1 mg of protein extract from cancer and normal tissues, in order to compensate for the low abundance of some proteins and circumvent the impact of dyes on MS identification. After matching with 2D-DIGE images using imagemaster software (GE Healthcare, Velizy Villacoublay, France), the protein spots were localized on the replicate gels and excised. The peptides produced by tryptic digestion of spots were analyzed by MALDI-TOF MS, and all proteins were successfully identified by peptide mass fingerprinting (Table 2).

Among them, aminoacylase-1, pre-mRNA-processing factor 19, T-complex protein 1 subunit α and T-complex protein 1 subunit β (TCP1β) are reported for the first time to be differentially expressed between tumor and normal colon mucosa. This points to the fact that the differential expression profile of CRC tissue has still not been fully characterized, despite the growing number of proteomic analyses. However, our data also include several proteins that were reported in previous publications, such as α-enolase [11,13,21–23], tropomyosin β-chain [24–26], and HSP60 [21,23,27]. This indicates that our 2D-DIGE analysis was accurate and in concordance with prior studies. After the identification of potentially interesting tissue markers, it was necessary to go further, first confirming differential expression with independent techniques, and then extending observations made in tissue to serum. These are the steps called marker qualification by Rifai *et al.* [18].

### Marker qualification

To confirm the differential expression results obtained by 2D-DIGE analysis, western blot was carried out for eight proteins among 17, with tissue samples from eight independent patients (Table 1; Fig. 2C). HSP60, glutathione-*S*-transferase pi (GST-Pi), α-enolase, TCP1β and cytokeratin 19 (CK19) were detected in the large majority of the samples, and significant overexpression in tumor tissue as compared with matching normal mucosa was confirmed for the first four proteins, but not for CK19. For phosphoglycerate mutase 1 (PGAM1) and HSP90β, the overall signal level was low, so it was difficult to draw conclusions. Leukocyte elastase inhibitor (LEI) was detected in only four of eight patients, but when detected it was consistently less abundant in colon carcinoma than in normal tissue, in agreement with our 2D-DIGE results but in contrast to published data [28]. Taken these findings together, there was good concordance between western blot data and 2D-DIGE results.

The aim of our study was to identify a serological candidate biomarker for CRC, as blood-based tests are easier to implement in routine clinical practice. Among potential CRC markers confirmed by western blot, HSP60 had the ability to reach the bloodstream. It is actively secreted by tumor cells [15], and has been found in plasma of individuals with cardiovascular disease risk [29,30]. Moreover, HSP60 was identified as one of the proteins with the highest fold change ratio (3.25, *P* < 0.0001) in 2D-DIGE between colonic tumors and matching normal mucosa. For all of these reasons, we focused on HSP60 for marker qualification in serum.

### Confirmation of HSP60 overexpression in colonic adenocarcinoma by immunohistochemistry (IHC)

**Fig. 3:**
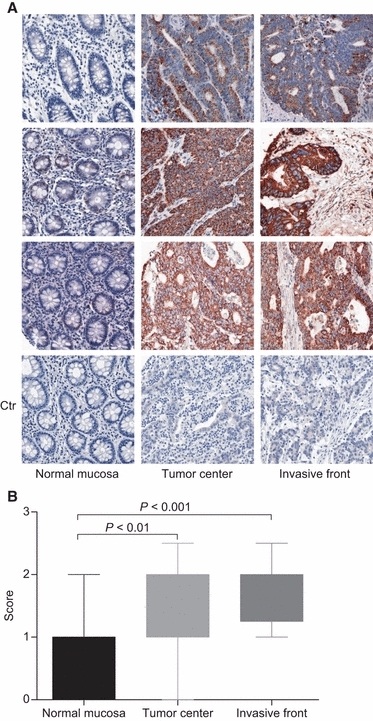
(A) Representative immunohistochemical staining images of HSP60 in normal colonic mucosa, tumor center, and tumor just behind the invasive front; magnification, × 20. For negative controls (bottom panels, Ctr), primary antibody against HSP60 was replaced by an irrelevant mouse IgG. (B) Comparison of HSP60 staining scores between matched normal mucosa, invasive front and tumor center in a series of 20 specimens from CRC patients. Analysis of variance with Friedman's test showed significant differences in the dataset (*P* < 0.0001). Pairwise *post hoc* comparisons were performed with Dunn's multiple comparison test, and the corresponding *P*-values are shown.

Immunohistochemical analysis was performed to control the mAbs against HSP60 selected for immunoassay development and to check their immunoreactivity profiles. The additional aim of IHC was to further confirm 2D-DIGE data with another independent technique. To this end, 20 independent colon cancer tissue specimens were selected from archived formalin-fixed, paraffin-embedded tissue blocks. Clinical and pathological data of corresponding patients are shown in Table 3. For each patient, matched tissue samples corresponding to the tumor center, tumor just behind the invasion front and adjacent normal mucosa were analyzed. Representative immunohistochemical images obtained with the mAb 11D5E10 are shown in Fig. 3A. As expected, HSP60 immunostaining was mainly cytoplasmic in epithelial cells [16,31]. It had a particulate appearance, consistent with mitochondrial localization. Many of the normal colonic mucosa specimens had no staining, and some exhibited weak positive reactivity to HSP60, whereas CRC tissues showed moderate to strong reactivity. Overall, HSP60 intensity was significantly stronger in the invasive front (1.7 ± 0.5, *P* = 0.0006) and tumor center (1.5 ± 0.7, *P* = 0.0045) than in normal mucosa (0.7 ± 0.6) (Fig. 3B). Very similar results were also obtained with mAb 16F11D12 (data not shown), suggesting that both antibodies are suitable for immunoassay development. Moreover, these results confirm overexpression of HSP60 in CRC tissue, in agreement with our 2D-DIGE and western blot data, as well as with published IHC series that analyzed HSP60 expression in CRC tissue [32,33].

**Tale 3:** Clinical data of colon cancer patients used in IHC analysis. UICC, Union for International Cancer Control

	*n*	%
Sex		
Male	10	50
Female	10	50
Tumor localization		
Right colon	7	35
Left colon	7	35
Transverse colon	3	15
Sigmoid	3	15
Global staging (UICC)		
I	1	5
II	5	25
III	12	60
IV	2	10

### Analytical method validation for HSP60 ELISA on VIDAS

To compare HSP60 levels in sera of CRC patients and healthy individuals, we set up a prototype HSP60 sandwich ELISA assay on the VIDAS immunoassay platform, using mAbs 11E5D10 and 16F11D12. Before moving to the next phase of our study, which was marker qualification in serum [18], a preliminary and partial analytical evaluation of the HSP60 ELISA prototype was carried out. Aspects of the clinical performance of a biomarker, such as sensitivity and specificity, are also impacted by the analytical performance of the assay that is used for its measurement. Consequently, it is of utmost importance to use validated methods in order to generate robust and reproducible data. Method evaluation protocols were simplified from the cognate Clinical and Laboratory Standards Institute guidelines, mainly by lowering the number of repeat measurements.

To establish the calibration model, seven nonzero standard points spanning an assay range of 0.5–20 ng·mL^−1^ were used. These calibrator points were assayed in duplicate in five consecutive runs, and the concentration–signal relationship was modeled with the four-parameter logistic function. The goodness of fit for repeated standard curves was analyzed by using *r*^2^, and was equal to 0.999, indicating a nearly perfect correlation. The appropriateness of the model was evaluated by calculating the percentage of relative error (RE) for back-calculated calibrator points (Table 4). The absolute values of RE were between 0.2% and 11.2%. The coefficients of variation (CVs) of calibrator point replicates were between 4.4% and 10.3%. As both of these acceptability criteria were lower than the recommended limit of 15% [34], the calibration model was deemed to be acceptable. Assay precision was assessed with six CRC serum samples that had low, medium and high HSP60 levels. For reproducibility (total precision), %CV varied between 4.8% and 15.6%. As expected, within-run precision was the main contributory factor to total variability (Table 5).

**Table 4:** %REs and %CVs of back-calculated standard curve values of HSP60 ELISA assay

Calibrator point	A	B	C	D	E	F	G
Nominal value (ng·mL^−1^)	0.5	1.0	2.5	5.0	7.5	10.0	20.0
Mean back-calculated value (ng·mL^−1^)	0.6	1.0	2.5	4.9	7.4	10.2	20.0
%RE	11.2	2.3	0.4	1.2	1.4	1.6	0.2
%CV	10.3	5.8	4.7	6.7	5.8	4.4	4.4

**Table 5:** HSP60 ELISA assay precision

Sample	QC1	QC2	QC3	QC4	QC5	QC6
Mean dose (ng·mL^−1^)	0.6	2.3	4.4	8.8	13.8	20.2
%CV intra-assay	13.3	5.9	3.3	7.2	5.3	4.4
% Variation part^a^	73	83	44	57	88	86
%CV inter-assay^b^	15.6	6.4	5.0	9.5	5.6	4.8

a Percentage of total variability attributable to intra-assay precision.

b Total variability, all assessed sources (intra-assay, run, day, instrument). QC: quality control sample.

The limit of blank (LOB) was determined on 42 replicate measurements of a blank serum sample. The LOB is the 95th percentile of the distribution of concentrations calculated from the standard curve: it was 0.12 ng·mL^−1^. The limit of detection (LOD) is the lowest concentration of the biomarker that the designed assay can reliably differentiate from the background noise. Four replicates of four serum samples, with HSP60 levels between LOB and four-fold LOB, were tested for 3 days. The LOD was 0.30 ng·mL^−1^. Two of the samples previously used for LOD determination were tested again in four replicates in two independent runs, in order to assess the lower limit of quantification (LLOQ). The LLOQ is the lowest concentration that can be measured with acceptable accuracy and precision. The LLOQ was 0.30 ng·mL^−1^, like the LOD. All of these analyses show the satisfactory analytical performances of our prototype and guarantee the quality and reproducibility of results obtained using this assay.

### Qualification and verification of HSP60 as a serum biomarker of CRC

**Fig. 4:**
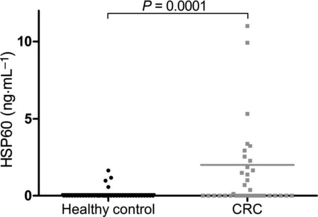
Serum levels of HSP60 in the qualification cohort, 40 healthy controls and 40 CRC patients, measured by ELISA. Serum HSP60 levels were significantly elevated in CRC patients (*P* = 0.0001, one-tailed Mann–Whitney test). The gray line represents the mean HSP60 concentration for the CRC group.

The qualification cohort (cohort I) comprised 40 CRC patients and 40 healthy individuals; their clinical data are presented in Table 6. Mean HSP60 levels measured by ELISA in these control and cancer sera were 0.1 ± 0.1 and 2.0 ± 0.6 ng·mL^−1^, respectively. This increase in HSP60 levels in CRC patients was statistically significant (*P* = 0.0001; Fig. 4), indicating that HSP60 is a potential serum biomarker for CRC.

**Table 6:** Clinical data of CRC patients and controls assayed by ELISA. UICC, Union for International Cancer Control

	Cohort I	Cohort II
Age (years)		
Control	55 ± 5	58 ± 4
Cancer	71 ± 11	70 ± 11
Sex, male/female, no. (%)		
Control	27 (68)/13 (32)	54 (60)/36(40)
Cancer	25 (63)/15 (38)	61 (54)/51(46)
Tumor localization, no. (%)		
Right colon	10 (25)	40 (36)
Left colon	9 (23)	40 (36)
Transverse colon	1 (3)	6 (5)
Sigmoid	3 (8)	15 (13)
Rectum	17 (43)	11 (10)
Global staging (UICC), no. (%)		
I	8 (20)	27 (24)
II	8 (20)	29 (26)
III	15 (38)	29 (26)
IV	9 (23)	27 (24)

**Fig. 5:**
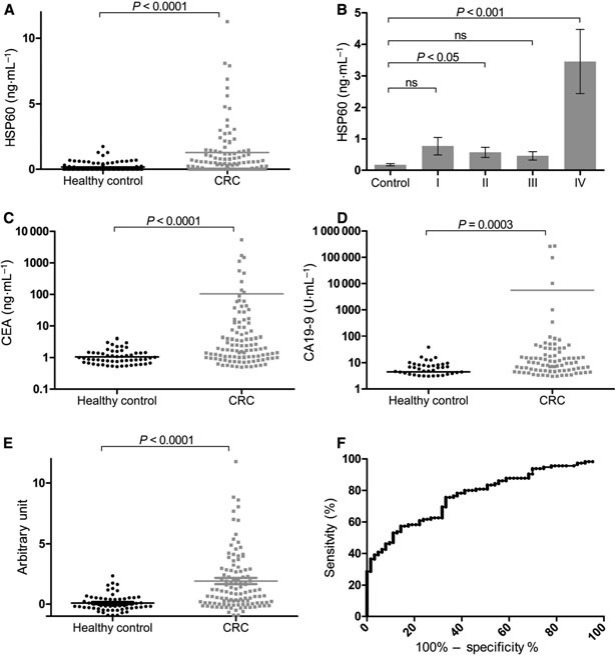
Serum levels of HSP60 and other CRC markers in the verification cohort. (A) HSP60, *n* = 202, AUC = 0.70. (C) CEA, *n* = 175, AUC = 0.70. (D) CA19-9, *n* = 175, AUC = 0.65. (E) Three-marker combination calculated with a logistic regression model, expressed in arbitrary units, *n* = 175. Mean marker concentrations are represented by lines. Control and cancer groups were compared by use of the one-tailed Mann–Whitney test. (F) Receiver-operating characteristic curve of the three-marker combination, AUC = 0.77. (B) HSP60 concentration according to CRC stage (I–IV). Data are means ± standard errors. Analysis of variance with Friedman's test indicated significant differences in HSP60 levels between groups (*P* < 0.0001). Pairwise comparisons were performed with Dunn's multiple comparison test, and the corresponding *P*-values are shown.

To verify the increase in HSP60 serum levels observed in CRC patients, a second and independent cohort of 90 healthy donors and 112 CRC patients was assayed (cohort II). This verification cohort was designed so that each clinical stage of the disease was equally represented among the CRC patients (Table 6). Again, serum HSP60 levels were significantly higher in CRC patients (1.3 ± 0.3 ng·mL^−1^; range, 0–25 ng·mL^−1^) than in healthy volunteers (0.2 ± 0.1 ng·mL^−1^; range, 0–1.7 ng·mL^−1^) (*P* < 0.0001) (Fig. 5A), confirming the observation made on the first cohort. Among CRC patients, only 38% had an HSP60 level < 0.30 ng·mL^−1^, which is the lower limit of quantification, as compared with 70% for healthy controls. Given the concordant results in two independent cohorts and the robust analytical performance of our HSP60 ELISA assay on VIDAS, we can confidently conclude that serum HSP60 levels are more frequently increased in CRC patients than in healthy controls.

The clinical performance of serum HSP60 as a biomarker to discriminate between cancer and noncancer patients was assessed with receiver-operating characteristic curve analysis. The area under the curve (AUC) represents an average of the sensitivity over all possible specificities. For the verification cohort (*n* = 202), the AUC was 0.70 [95% confidence interval (CI) 0.63–0.77]. When the specificity was set at 90%, the sensitivity of HSP60 was 40%.

We also analyzed whether serum HSP60 levels were increased at all clinical stages of disease. Figure 5B shows that this rise was mainly observed in patients with stage IV cancer, the mean level in this group reaching 3.5 ± 1.0 ng·mL^−1^. Moreover, samples below the limit of quantification were less frequent in this group (26%). These results imply that HSP60 is a serum marker for advanced stages of disease, and that it may not be well suited for early detection.

### Comparison and combination of HSP60 with current serum biomarkers of CRC

Serum CEA and CA19-9 are used in clinical practice for CRC patient monitoring, and contribute to diagnosis. CEA and CA19-9 levels were tested in the verification cohort with commercial assays. As expected, CEA levels were significantly higher in CRC patients (104 ± 52 ng·mL^−1^) than in healthy volunteers (1.1 ± 0.1 ng·mL^−1^) (*P* < 0.0001) (Fig. 5C). CA19-9 levels were also significantly higher in CRC patients (5592 ± 3359 U·mL^−1^) than in healthy volunteers (4.5 ± 0.8 U·mL^−1^) (*P* = 0.0003) (Fig. 5D). The AUC values were 0.70 (95% CI 0.62–0.78) and 0.65 (95% CI 0.57–0.73) for CEA and CA19-9, respectively. When the specificity was set at 90%, the sensitivities of CEA and CA19-9 assays reached 41% and 36%, respectively. These results indicate that the diagnostic performance of serum HSP60 for cancer/no cancer discrimination is very similar to that of CEA and better than that of CA19-9. Consequently, with the current assay format, HSP60 alone would be of limited clinical utility for the diagnosis of CRC, like CEA or CA19-9.

Combined use of markers can often improve clinical performance, as the types of biological information provided by the different markers do not totally overlap. We performed logistic regression to establish a mathematical model that combines HSP60, CEA, and CA19-9. Its output is expressed in arbitrary units; as expected, values in CRC patients were significantly higher than in healthy volunteers (*P* < 0.0001) (Fig. 5E). The AUC of the model reached 0.77 (95% CI 0.70–0.84) (Fig. 5F), showing a significant 7% improvement over the performances of individual markers. Similarly, when the specificity was set at 90%, the sensitivity of the three-marker combination increased to 47%. When the specificity was set as high as 98%, to be comparable with the reference standard fecal occult blood test assay Hemoccult II, the three-marker combination reached a sensitivity of 36%, in the range of sensitivity of Hemoccult II for cancer (25–38%) [35], but not better. However, the utility of this three-marker combination should be further evaluated for monitoring purposes, as it may represent an improvement over CEA or CA19-9 alone.

## Discussion

The aim of our study was to identify and verify new serum markers of CRC, as well as to generate data that will allow the best-suited clinical use to be chosen. To circumvent the well-known difficulties associated with direct protein biomarker discovery in serum [18], we carried out a comparison of protein expression levels, using 2D-DIGE, in paired tumor tissue and matching normal mucosa samples. 2D-gel electrophoresis analyses are often used on protein extracts from crude tissues [11]. However, tumor tissues are heterogeneous, and an enrichment step that allows partial or total purification of tumor cell populations from surrounding undesired cells may increase the significance of differential analysis results. Various methods can be used to achieve this, such as macrodissection [13], or laser capture microdissection, which is much more powerful but requires specific equipment [36]. As CRC is an adenocarcinoma, we isolated the epithelial cell population by using a kit based on magnetic beads coated with an antibody that recognizes two membrane antigens expressed on most normal and neoplastic human epithelial cells [37]. This easy method works well; no contaminant proteins, such as serum albumin, serotransferrin, or apolipoprotein AI, were found in our differential analysis. Among 800 protein spots present in our 2D-DIGE gels, we identified only 17 as being different between tumor and normal epithelial cells. This is comparable with other studies that analyzed pairs of CRC and normal tissue using 2D electrophoresis coupled to MS [13,21,22,25,28,38–40]: the number of proteins reported to be differentially expressed ranged from nine [40] to 52 [28]. For studies relying on the 2D-DIGE technique, this number was often higher than 30 [13,21,28], probably because of the gain in reproducibility resulting from the use of fluorescent dyes over more traditional, silver nitrate staining methods. In comparison, the number of differentially expressed proteins that we have found is lower. We suggest that this is because we analyzed purified cell populations rather than bulk tissue.

In this study, we identified 17 proteins as showing substantial differences between tumor and normal colon tissues. Differential expression of aminoacylase-1, pre-mRNA-processing factor 19, T-complex protein 1 subunit α and TCP1β has been, to our knowledge, shown for the first time (Table 2). Our data also included proteins reported in previous proteomic studies, such as α-enolase [11,13,22,23,41], tropomyosin β-chain [24–26], cytoplasmic actin 1 [21,27], GST-Pi [26,28], and HSP60 [21,23,27]. For these candidate biomarkers, our results were in agreement with published data. LEI (SERPINB1) was downregulated in tumor tissues, as shown by 2D-DIGE and further confirmed by western blot, unlike what has been reported by others [28]. This latter technique also showed that LEI was detected only in half of the patients (four of eight); the other half did not express LEI at all, at least not at levels that can be detected by western blot. This heterogeneity in expression levels could account for the contradictory results that are reported. For GST-Pi and TCP1β, there was good concordance between 2D-DIGE results and western blot; these proteins were frequently detected in our experiments in colon tissue, and should be further evaluated as tumor markers. For PGAM1 and HSP90β, although there was concordance between 2D-DIGE and western blot data, the tissue levels of these proteins were at the lower detection limit of both techniques, and more sensitive techniques, such as IHC, could be more suited for marker qualification. Finally, our proteomic data also indicate that S100A8 protein is more abundant in colonic tumors than in matched normal tissue, in agreement with 2D-DIGE data reported recently by Kim *et al.* [13]. Strikingly, we did not identify S100A9 as a differentially expressed protein, although it has been reported much more frequently than S100A8 [13,23,28,42]. Moreover, the study by Kim *et al.* [13] also showed that the levels of both S100A8 and S100A9 are increased in plasma of CRC patients, indicating that they could be interesting serological markers for CRC.

As the number of studies dealing with the differential expression profiles of CRC tissue increases, a large collection of potential candidate markers are becoming available. Nevertheless, each study brings its own discrepancies, resulting from methodological differences in sample collection, processing, or analysis, and from variations in genetic or pathological characteristics of the patients enrolled. To generate reliable data that will lead to the validation and clinical use of new biomarkers, it is necessary not only to confirm observations with independent techniques, but also to work on well-characterized patient samples and increase the number of patients included in the analyses. For these reasons, we used four different protein detection techniques and patient cohorts from four different sources in our study.

For the next phases of biomarker discovery and validation, which are marker qualification in serum and further verification [18], we uniquely focused on HSP60 in our study. Both our results and data from the literature suggest that it has the potential to be a serum biomarker, in addition to being a tissue biomarker [32]. Indeed, HSP60 is actively secreted by tumor cells, most probably through the exosomal pathway [15], and titers of antibodies against HSP60 were reported to be higher in CRC patients than in controls [43]. None of the commercial HSP60 assays that we evaluated had a satisfactory precision and detection limit in serum; and we therefore set up an in-house assay method. With %CVs for total precision in the range of 5–15%, our HSP60 assay was extremely reliable for the detection of serum HSP60, and allowed us to show clearly that HSP60 itself was a serum marker for CRC in two independent cohorts. The initial assessment of the diagnostic performance of the marker showed 40% sensitivity at 90% specificity, which is very similar to the performance of CEA and better than that of CA19-9. Our data did not provide support for a clear correlation between the serum levels of HSP60 and the global staging of cancer, even though HSP60 levels were significantly higher in stage IV patients than in other groups. This was somewhat unexpected, because such a correlation has been shown in tissue with the use of independent techniques [16,17]. However, events observed in cancer tissue are not always confirmed in distal fluids such as serum, this being among the main difficulties of carrying out marker discovery in tissue rather than directly in the target fluid [18]. In colonic tissue, the increase in HSP60 expression is initiated early during carcinogenesis; it has even been reported to occur in preneoplastic lesions [32]. This suggests that HSP60 could be of interest for screening and early detection of CRC. Unfortunately, the ELISA data that we generated failed to support this hypothesis. In the verification cohort (cohort II), the difference in mean serum HSP60 concentration between stage I patients and healthy controls did not reach statistical significance, and the difference was barely significant between stage II patients and controls (*P* < 0.05). Serum HSP60 levels were higher in stage IV patients than in all other groups (Fig. 5B), reminiscent of CEA. As a consequence, serum HSP60 seemed to be more useful for prognosis and monitoring purposes than for screening or early detection of CRC. However, a limitation regarding this conclusion stems from the analytical limits of our HSP60 ELISA, which has a lower quantification limit of 0.3 ng·mL^−1^. Among CRC patients, 38% had serum HSP60 levels below this limit, suggesting that the marker may benefit from an assay method with increased analytical sensitivity that is able to quantify in the dozens of pg·mL^−1^ range.

An increase in HSP60 expression as compared with normal tissue has been shown for a variety of tumors, including Hodgkin's lymphoma, and prostate, ovarian and breast adenocarcinomas (reviewed in [44]). At least for some of these cancers, serum HSP60 levels might be associated with the presence or progression of cancer, as we have shown for CRC. Furthermore, HSP60 is a key factor involved in inflammation, and serum HSP60 levels might also be increased in patients with inflammatory pathologies such as Crohn's disease and ulcerative colitis [45]. Further studies are needed to determine the serum HSP60 levels in these populations, to obtain a sound understanding of how serum HSP60 can be used to contribute to the prognosis or monitoring of CRC patients.

## Experimental procedures

### Patients and specimens

Colonic adenocarcinoma and matching normal mucosa were obtained from 23 patients who underwent surgical resection. Normal mucosa was taken from the surgical margins, at least 10 cm away from the tumor, and was pathologically certified to be normal mucosa. Each patient gave informed, written consent, and the sampling protocol was in accordance with good clinical practice. All tissues were collected in RPMI, immediately frozen in the pathology laboratory after resection, and stored at −80 °C until use.

Serum samples from 152 patients diagnosed with CRC and 130 healthy volunteers were collected for the study. CRC samples were obtained from academic hospitals in Lyon, Dijon and Saint-Etienne (France), and control samples were obtained from blood donors at Etablissement Français du Sang, the French blood bank. Cohort I included 40 CRC patients and 40 controls used for marker qualification, and cohort II included 112 CRC patients and 90 controls used for marker verification.

### 2D-DIGE

Tissues were cut into small pieces, and were treated in a Medicon (Dako, Hamburg, Germany) to generate a cell suspension. Epithelial cells were separated from other cell types present in tissue with the Dynabeads Epithelial Enrich kit (Invitrogen, Cergy Pontoise, France), which targets EpCam membrane antigen, and suspended in water containing 0.9% NaCl and protease inhibitors (Roche Diagnostics, Meylan, France). Cell lysis and protein extraction were carried out in lysis buffer (7 m urea, 2 m thiourea, and 4% Chaps), with two cycles of sonication and freezing. After centrifugation at 40 000 ***g*** for 30 min, the protein content of the extract was determined with the Bio-Rad Protein Assay kit (BioRad, Marnes la Coquette, France).

Protein labeling was carried out on 50 μg of each tumor and matching normal mucosa extracts with Cy3 and Cy5 fluorescent dyes. Caco-2 cell extract, used as an internal standard, was labeled with Cy2 dye. According to the user guide, a ratio of 400 pmol of fluorescent dye per 50 μg of protein extract was used (GE Healthcare, Velizy Villacoublay, France). The labeling reaction was performed at 4 °C for 30 min, and quenched with 1 μL of lysine (10 mm) for 10 min on ice, in the dark. For each patient, 50 μg of tumor and control extracts, labeled with different dyes, was pooled with 50 μg of Cy2-labeled internal standard, and was focused with immobilized pH gradient (IPG) strips (Ready Strip pH 5–8, 17 cm; BioRad) on an IEF Cell apparatus (BioRad). A dye-swap replicate was also used. Following isoelectrofocalization, IPG strips were washed with 50 mm Tris/HCl equilibration buffer containing 2% dithiothreitol for 15 min, and then washed again with the same buffer containing 2.5% iodoacetamide for 15 min. SDS/PAGE was carried out for the second dimension, using 7.7–16.5% gradient polyacrylamide gels, at 40 mA per gel, for 5 h. Labeled proteins in each gel were visualized with a ProXpress (Perkin Elmer, Courtaboeuf, France) fluorescence scanner at 488/600 nm for Cy2, 532/580 nm for Cy3, and 633/520 nm for Cy5.

Scanned gel images were analyzed with image master 2d-platinium 6.0 (GE Healthcare). The best internal standard image was used as the master reference. The protein spots on the other internal standard gel images were matched with the master reference to ensure that the same protein patterns were compared between gels. Spot volumes measured on Cy3 and Cy5 gels were transformed in logarithm base 2 and normalized by dividing each Cy3 or Cy5 spot volume by the corresponding Cy2 (internal standard) spot volume. Abundance changes were calculated for each paired tumor and control sample, and compared by the use of Wilcoxon matched-pairs test.

### MALDI-TOF MS

For each patient, a replicate 2D electrophoresis gel was run with 1 mg of protein extract from cancer and adjacent normal tissue, stained with Simply Blue (Invitrogen), and then matched with the 2D-DIGE gel maps. Protein spots were excised from 2D electrophoresis gels and digested in-gel with trypsin with the automated ProteineerSP and ProteineerDP robots (Bruker Daltonics, Wissembourg, France), following the protocols of the manufacturer. Digests were transferred automatically by thin-layer preparation on an AnchorChip MALDI sample plate, with an α-cyano-4-hydroxycinnamic acid matrix. MS spectra were recorded in the positive reflectron mode of an Ultraflex TOF/TOF MALDI-TOF mass spectrometer (Bruker Daltonics). The external calibration of MALDI mass spectra was carried out with the singly charged monoisotopic peaks of Bruker's peptide mixture. To achieve mass accuracy, internal calibration was also performed with the peptides resulting from the autolysis of trypsin. The peptide mass profiles obtained by MALDI-TOF MS were analyzed with proteinscape 1.3 (Bruker Daltonics), using mascot 2.0 (MatrixScience, London, UK) for peptide mass fingerprinting. Observed peptide masses were compared with the theoretical masses derived from the sequences contained in the SWISS-PROT online database. The search parameters used were as follows: carbamidomethylation for cysteines, oxidation, peptide mass tolerance of maximum 50 p.p.m. allowed, and a maximum of one missed enzymatic cleavage. The species of origin was restricted to human.

### Western blot

Protein extraction from tissue samples was carried out as for 2D-DIGE. SDS/PAGE was performed with 4–12% Bis-Tris NuPage gels (Invitrogen); 10 μg of protein extract from each patient was loaded per lane. Following electrophoretic separation, proteins were transferred onto poly(vinylidene difluoride) membranes, stained with amidoblack, and incubated for 1 h with antibodies diluted in blocking buffer (5% nonfat dry milk, 15 mm Tris, pH 8, 140 mm NaCl, 0.5% Tween-20). Antibodies directed against HSP60 (clone 11D5E10), GST-Pi (clone 2D1G1) and LEI (clone 21B10A5) were obtained in-house and used at a concentration of 10 μg·mL^−1^. Antibodies against α-enolase (sc-100812), PGAM1 (sc-130334), TCP1β (sc-28556) and HSP90β (sc-69703) were from Santa-Cruz Biotechnology (Heidelberg, Germany), antibody against α-tubulin (clone 17H11) was from Rockland Immunochemicals (Gilbertsville, PA, USA), and antibody against CK19 (61010) was from Progen (Heidelberg, Germany). Commercial antibodies were assayed at a dilution of 1 μg·mL^−1^. After three washes with blocking buffer, membranes were incubated with horseradish peroxidase-conjugated anti-(mouse IgG) (Jackson ImmunoResearch, Newmarket, UK). Chemoluminescent substrate was from Thermo Scientific (Super Signal West Dura Extend Duration Substrate), and membranes were scanned with a VersaDoc system (BioRad).

### IHC

A small tissue microarray (TMA) was constructed with archived formalin-fixed, paraffin-embedded tissue blocks from 20 colon cancer patients. For each patient, three 1.5-mm biopsy cores from the center of the tumor and three from the invasion front were retrieved and inserted in a recipient paraffin block. Similarly, three cores from matching normal colon mucosa were collected and added to the TMA block. Sections 4 μm thick were cut from the TMA block and transferred to Superfrost slides (Menzel Glaser, Braunschwrig, Germany), dewaxed with three baths of toluene, and gradually rehydrated in alcohol/water baths with decreasing alcohol content. Antigen retrieval was carried out in 0.01 m (pH 6) citrate buffer for 30 min, at 98 °C. Endogenous peroxidase activity was blocked with 3% hydrogen peroxide for 5 min. HSP60-specific mAbs 11D5E10 and 16F11D12, generated in-house, were diluted to 5 μg·mL^−1^ with the background reducing dilution buffer (Diagnostic BioSystems, Pleasanton, CA, USA), and were incubated at room temperature for 1 h. Detection was carried out according to the manufacturer's instructions, using the streptavidin–biotin-amplified Multilink kit (Biogenex, Fremont, CA, USA); the chromogen amino-3-ethyl-9-carbazole was incubated for 8 min. For nuclear counterstaining, the slides were treated with hematoxylin for 2 min.

TMA slides were digitized at × 20 magnification with the Scanscope scanner (Aperio Technologies, Oxford, UK). Virtual slides were examined by a pathologist on a computer with imagescope (Aperio Technologies). For each patient, HSP60 staining in the epithelial cells of the invasion front, the center of the tumor and normal mucosa was evaluated. The scoring was based on staining intensity, and results from triplicate cores were averaged. Imuunohistochemical staining was graded as negative (0), weakly positive (1), moderately positive (2), and strongly positive (3).

### Fluorescent ELISA

ELISA was performed on a VIDAS instrument, an automated immunoassay system (bioMérieux, Marcy l’étoile, France). The solid phase receptacle, which serves as both a solid phase and a pipetting device, was coated with the capture mAb 11D5E10 at 30 μg·mL^−1^. The biotinylated detection antibody 16F11D12 was used at a concentration of 1 μg·mL^−1^. Buffers from a commercial VIDAS assay strip (Cat. No. 30315, bioMérieux) were used as described in the package insert, without additional changes. A hundred microliters of each control, standard and serum sample was directly added to well no. 2 of the VIDAS strip containing the conjugate buffer. To lower the limit of detection, assays were run with the long version of the assay protocol. Assay of CEA and CA19-9 levels in serum samples was carried out with commercially available VIDAS CEA (S) and VIDAS CA19-9 kits (bioMérieux), following the protocol provided by the manufacturer.

### Method validation

Standard points were prepared with serial dilutions of a recombinant HSP60 protein in a pool of control sera. To generate the data that were used to fit the master calibration curve, standard points were assayed in duplicate in five consecutive runs. A four-parameter logistic function was used to fit these data. The accuracy of standard curves was estimated by using *r*^2^ for goodness of fit and %RE for each standard point. %RE was calculated as follows: [(back-calculated value − expected nominal value)/expected nominal value] × 100%. Current guidelines recommend *r*^2^ > 0.99 and RE ≤ 15% for standards other than at the lower limit of quantification [46].

Assay precision was assessed in a combined repeatability and reproducibility experiment, with six sera that were evenly distributed within the calibration range. Each sample was tested in duplicate in each run; two runs per day and per instrument were carried out on two instruments for three consecutive days. A nested analysis of variance was performed on interpolated concentrations.

To determine the LOB, a blank sample, chosen according to the definition in the Clinical and Laboratory Standards Institute EP17-A guideline, was assayed 42 times. The LOB was defined as the 95th percentile of the distribution of blank doses interpolated from the standard curve. The LOD was estimated with four serum samples with low HSP60 concentration (LOB < concentration < 4 × LOB), tested as four replicates, for 3 days. The standard deviation (SD) of this dataset was calculated in terms of dose, and the LOD was defined as LOB + cSD, where *c* = 1.645/(1 − 1/4*f* ), *f* being the degrees of freedom of SD. The LLOQ was estimated with two of the samples used for LOD assessment, with concentrations as close as possible to the LOD, and corresponds to the lowest reliable concentration that fulfils the accuracy expectation (RE ≤ 15%).

### Statistical analyses

All statistical analyses were performed with graphpad prism 5.0 or sas V9. A *P*-value of < 0.05 was considered to be statistically significant.

## Abbreviations

AUC: area under the curve
CA19-9: carbohydrate antigen 19-9
CEA: carcinoembryonic antigen
CI: confidence interval
CK19: cytokeratin 19
CRC: colorectal cancer
CV: coefficient of variation
2D-DIGE: two-dimensional difference gel electrophoresis
GST-Pi: glutathione-*S*-transferase Pi
HSP: heat shock protein
IHC: immunohistochemistry
IPG: immobilized pH gradient
LEI: leukocyte elastase inhibitor
LLOQ: lower limit of quantification
LOB: limit of blank
LOD: limit of detection
PGAM1: phosphoglycerate mutase 1
RE: relative error
SD: standard deviation
TCP1β: T-complex protein 1 subunit β
TMA: tissue microarray
